# The Interplay between the Unfolded Protein Response, Inflammation and Infection in Cystic Fibrosis

**DOI:** 10.3390/cells10112980

**Published:** 2021-11-02

**Authors:** Pascal Trouvé, Claude Férec, Emmanuelle Génin

**Affiliations:** Inserm, Univ Brest, EFS, UMR 1078, GGB, F-29200 Brest, France; claude.ferec@univ-brest.fr (C.F.); emmanuelle.genin@inserm.fr (E.G.)

**Keywords:** cystic fibrosis, unfolded protein response, inflammation, infection

## Abstract

In cystic fibrosis (CF), p.Phe508del is the most frequent mutation in the Cystic Fibrosis Transmembrane conductance Regulator (*CFTR*) gene. The p.Phe508del-CFTR protein is retained in the ER and rapidly degraded. This retention likely triggers an atypical Unfolded Protein Response (UPR) involving ATF6, which reduces the expression of p.Phe508del-CFTR. There are still some debates on the role of the UPR in CF: could it be triggered by the accumulation of misfolded CFTR proteins in the endoplasmic reticulum as was proposed for the most common CFTR mutation p.Phe508del? Or, is it the consequence of inflammation and infection that occur in the disease? In this review, we summarize recent findings on UPR in CF and show how infection, inflammation and UPR act together in CF. We propose to rethink their respective role in CF and to consider them as a whole.

## 1. Introduction

Cystic fibrosis (CF) is an autosomal recessive disease caused by mutations in the *Cystic Fibrosis Transmembrane Conductance Regulator* (*CFTR*) gene [1]. To date, 2106 mutations have been identified in the *CFTR* gene, some resulting in expression and/or function defects of the CFTR protein (http://www.genet.sickkids.on.ca/StatisticsPage.html, accessed on 25 October 2021). The CFTR protein belongs to the adenosine triphopsphate (ATP)-binding cassette (ABC) transporter’s family and mainly acts as a cAMP-regulated chloride (Cl^−^) channel at the apical membrane of epithelial cells [2,3]. In patients, the malfunction of the CFTR channel leads to the thickening of the secreted mucus because it is functionally linked to the epithelial sodium channel (ENaC) that is rate limiting for Na^+^ and fluid absorbing from the airway surface [2,4]. The thickening of the mucus promotes infections and inflammation [5,6]; ultimately, this leads to the progressive destruction of the airways which is the main cause of morbidity and mortality in CF patients. The gastrointestinal tract and the reproductive organs are also affected, but are treated symptomatologicaly.

The deletion of the phenylalanine residue at the position 508 (p.Phe508del) is the most common disease-causing CFTR’s mutation [7]. It leads to a misfolded protein which is retained within the endoplasmic reticulum (ER) and is rapidly degraded by the ER-associated degradation (ERAD) [6]. The p.Phe508del-CFTR protein remains in a core-glycosylated form in the ER and only a negligible quantity is expressed at the plasma membrane of the cells [6,7]. Although this is still subject to debate, the retention and/or the accumulation of the p.Phe508del-CFTR protein within the ER likely induces an ER stress leading to the triggering of the Unfolded Protein Response (UPR). The UPR is a normal physiological cellular recovery process aimed to regulate the protein load in the ER [8,9,10]; in CF, beside the unfolded protein retention in the ER, UPR is most probably triggered by inflammation and infections [11,12].

As mentioned above, the triggering of the UPR in CF is in debate. Some studies suggested that p.Phe508del-CFTR expression in primary airway cells does not activate the UPR in its typical form [13]. Nevertheless, we provided some lines of evidence indicating that the UPR is likely triggered in CF cells [14]. Our findings supported earlier results showing that the activation of the UPR inhibits endogenous CFTR expression [15,16]. Nevertheless, it is conceivable that high amounts of the p.Phe508del-CFTR protein are necessary to trigger the UPR. It is not known how the increased p.Phe508del-CFTR expression might affect the UPR but the overexpression of recombinant p.Phe508del-CFTR induces ER stress and activated the UPR [15,16]. Another evidence that UPR is present in CF cells is that the IRE1α mRNA levels are upregulated in freshly isolated CF human bronchial epithelial cells when compared to normal epithelial cells [17]. This increased expression is coupled to increased XBP-1 mRNA levels, without any induced overexpression expression of CFTR [17]. Whereas, the contribution of the IRE1α/XBP-1 branch of the UPR is proposed to be triggered by the inflammatory responses in the CF airway epithelia [18], IRE1α mRNA levels are higher in CF cells before the induction of inflammation [11]. Therefore, UPR is likely occurring before inflammation. In our opinion, the UPR is triggered in cells when p.Phe508del-CFTR retention exceeds a threshold. Below this threshold, ERAD is sufficient to protect the cells. Above this threshold, the UPR is triggered leading to the transcription of chaperons and ERAD proteins. The threshold of the amount of the p.Phe508del-CFTR protein could be reached when the ER is overloaded by other proteins such as inflammatory proteins or if ERAD is altered. This hypothesis is reinforced by the ratiometric sensing of BiP-client versus BiP levels [19,20].

Whatever triggers the UPR, whether it is the protein retention itself, inflammation, or both, it is observed that the UPR is triggered in CF cells and that therefore it must be taken into account in the pathophysiology of the disease [21]. Our aim is here to review what is known about UPR, inflammation and infection in CF, in order to show that they are not independent and that the triggering of the UPR is fully involved in the pathophysiology of CF. We propose that the UPR is as important as inflammation and infection in CF, and that the three of them should be considered at the same level because they interplay.

## 2. Misfolding and Unfolding of Proteins and Their Processing

**Figure 1:**
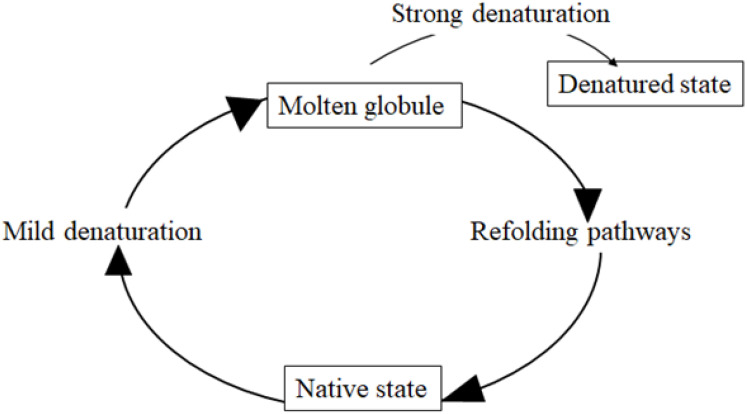
Molten globule. The molten globule is a protein state which is different from both the native state and the denatured state. It is an intermediate lacking the compaction of side chains as in the native state of a protein. Molten globules are collapsed and exhibit a native secondary structure with a dynamic tertiary structure. These features are that observed in the transient intermediates during the folding of globular proteins that present collapsed hydrophobic region.

Membrane and extracellular proteins are translated on the cytosolic surface of the ER and achieve their folding by acquiring their secondary structure according to the diffusion-collision theory [22,23]. During this process, a protein maintaining an early maturation step is defined as a misfolded protein. Misfolded proteins expose some residues to their surface that, in the normal state, are buried inside the proteins and shielded from the solvent. This leads to illegitimate protein–protein interactions and insoluble aggregation. Misfolded proteins mostly remain in folding intermediates called “molten globules” which are collapsed structures with some native-like secondary structure, augmented conformational flexibility, tertiary labile structure and dehydrated inside [24]. The molten globule state is a thermodynamic intermediate state, different from the native and from the denatured state [24] (Figure 1).

For their part, unfolded proteins exhibit higher compaction, formation of hydrophobic clusters and fluctuations of their secondary structure. Chaperones recognize proteins when they are in the molten globule state. The p.Phe508del mutation of the CFTR gene inhibits the last stage of the protein folding [25]; the protein is in the molten globule state, not allowing its trafficking through normal intracellular pathways and leading to its mislocalization and degradation [25,26]. Studies of the p.Phe508del-CFTR protein have shown that the altered protein folding with a global destabilization is likely wrong, and that a regional unfolding is likely occurring [27,28,29]; whereas, while misfolding and unfolding are different, they are supposed to trigger the same response.

To prevent harmful effects in the ER, aberrant proteins are recognized and degraded. In this context, wild-type (Wt) and p.Phe508del-CFTR protein, which both have inefficient processing, are rapidly degraded [30]. To ensure that proteins meet the quality criteria for their function, they pass through the ERQC which involves classical chaperones such as DnaK/Hsp70, DnaJ and GrpE, GroEL/Hsp60 and GroES and proteases [31]; they maintain proteins in a soluble form, allowing their accession to proteases. The ERQC proceeds at two levels: the general level and the protein-specific one. The general level is the primary quality control that applies to all proteins through the recognition of common structural features in non-native states. The protein-specific level or secondary quality control applies to individual proteins by the recognition of specific features by specialized chaperones. For glycoproteins such as CFTR, this secondary quality control is based on the recognition of specific glycosylation intermediates by the two lectin chaperones, calnexin and calreticulin [32,33].

When proteins do not reach their correct conformation, they are targeted by the ERAD which operates in several steps: substrate recognition, targeting, retrotranslocation to the cytosol, ubiquitinylation and proteasomal degradation [34]. Specific features of the nascent protein such as hydrophobic patches, N-linked glycans and disulphide bonds have to be present in the protein for substrate recognition by ERAD [35,36]. The first two recognition modes are involved in the maturation of CFTR.

Hydrophobic patch recognition involves molecular chaperones of the Hsp70 family and the immunoglobulin-binding protein (BIP/GRP78) [34]. Membrane proteins harbouring a large cytoplasmic domain such as CFTR bind to Hsp40s and Hsp70s, the latest facilitating the degradation of p.Phe508del-CFTR. The Hsp70-p.Phe508del-CFTR interaction is necessary to recruit the E3 ubiquitin ligase complex linked to BiP in the ER membrane that further ubiquitinylates and degrades CFTR [37,38,39,40].

## 3. The Unfolded Protein Response (UPR)

The accumulation of unfolded or misfolded proteins in the ER triggers the activation of complex pathways during the UPR [10,41]. The sensor of the UPR is BiP. IRE1, PERK and ATF6 are its effectors. The effect of UPR is the transcription of genes encoding molecular chaperones, folding catalysts, ERAD and antioxidant proteins. The UPR signaling also decreases the global protein synthesis in order to avoid an overload of the ER; it favors the cell survival, although the ER is overloaded by unfolded proteins or if UPR itself is altered, the apoptotic pathway is triggered.

### 3.1. The Binding Immunoglobulin Protein (BiP)

The ER resident chaperone BiP (glucose-regulated protein 78, GRP78) is member 5 (HspA5) of the Hsp A family (Hsp70) [42]. Under normal conditions, it is abundant in mammalian cells, representing about 5% of the lumenal content of the ER [43]. Its synthesis is further induced by stresses occurring in the ER [43]. BiP is a 654 amino acid protein composed of a conserved N-terminal nucleotide-binding domain (NBD) and of a substrate-binding domain (SBD), with a linker between them. The interaction of BiP with client proteins is regulated by its nucleotide-bound state [44]. It is a water-soluble protein with small hydrophobic patches involved in the recognition of unfolded proteins [45]. The binding of BiP onto its targets is regulated by at least seven different HSP40 co-chaperones (ERdj1-7), 2 nucleotide exchange factors and through reversible AMPylation [46,47]. When BiP binds its target, it releases the initially bound effectors of the UPR, which are in the membrane of the ER: ATF6, IRE1 and PERK [42]. Once unbound and activated, they decrease the translation of proteins and enhance correct folding. BiP also has a non-chaperon function; it chelates calcium in the ER and is found on the cell surface with a multifunctional receptor function [48].

### 3.2. ATF6 Signaling

ATF6 (90 KDa) is a transcription factor of the leucine zipper family. Its role in UPR is to initiate a transcriptional program to restore ER homeostasis by increasing BiP expression, promoting chaperones, regulating lipid synthesis, stimulating ER-degradation and enhancing N-glycosylation [10,49]. It is also involved in the UPR-related cell death. Once released from BiP, ATF6 is exported to the Golgi where it is successively cleaved by the serine protease 1 (S1P, MBTPS1) and the metalloprotease S2P, which also activates SREBP-1 and -2 [50,51]. A cleaved fragment of 50 kDa (~400 amino acids), corresponding to the cytosolic N-terminal portion of ATF6 is released. It is composed of a transcriptional activation domain (TDA), a bZIP domain, a DNA-binding domain and a nuclear localization signals, while in the nucleus, it induces the expression of the UPR genes [52]. ATF6 can modulate gene expression by interacting with other bZIPs, such as CREB, CREB3L3, sterol regulatory element-binding transcription factor 2, XBP1 and various other transcription factors. ATF6 induces the expression of XBP1 and CHOP to enhance UPR signaling or apoptosis, respectively [53]. ER chaperones often contain in their promoter regions a unique cis-acting element called the ER stress response element (ERSE), with a consensus sequence that is CCAAT-N9-CCACG; indeed, ERSE is necessary and sufficient for the transcriptional activation of the genes of ER chaperones [54,55]. Because the general transcription factor NF-Y constitutively occupies the CCAAT part of the ERSE, the binding of ER stress response factors needs to bind to the CCACG part. In humans, there are in fact two isoforms of ATF6 (ATF6α and ATF6β) [56]. They both have the CCACG sequence. However, ATF6β exhibits a poor transcription factor activity because its TAD is lacking eight important amino acids. Instead of being an UPR gene activator, it is rather an inhibitor by forming heterodimers with ATF6α [56]. In addition, the timing of ATF6α and β activation following ER stress is another important isoform-specific characteristic. It was shown that the activation of ATF6α occurs earlier than that of ATF6β. There is an initial strong activation of ATF6α and then a modulation to a weaker activation. The mechanism by which ATF6α and β can regulate the strength of ER stress response depends on their binding to ERSE [57]. Other ER stress transducers sharing sequence similarities with ATF6 possess a transcription-activation domain and a bZIP domain. These transcription factors are Luman, OASIS, BBF2H7, CREBH and CREB4 [58]. Whereas they have structural similarities with ATF6, they have different activating stimuli, tissue distribution and specialized functions in regulating the UPR.

### 3.3. IRE1 Signaling

In humans, the two inositol-requiring enzyme 1 (IRE1) paralogues (IRE1α and β) are encoded by the ER to nucleus signaling 1 and 2 genes (ERN1, ERN2), respectively [59]. The IRE1α and β isoforms share 39% sequence homology. Whereas IRE1α (often referred to IRE1) is ubiquitously expressed, the expression of IRE1β is mainly observed in the gastrointestinal tract and in the pulmonary mucosal epithelium [60,61]. Human IRE1 is a 977 amino acid protein (~110 kDa). Its dissociation from BiP triggers the oligomerization, the autophosphorylation and the activation of its cytosolic kinase domain [61,62]. The phosphorylation in the kinase domain (Ser724, Ser726 and Ser729) is necessary to activate the cytosolic RNase domain and to recruit the tumor necrosis factor receptor-associated factor 2 (TRAF2) and the JNK pathway [63]. The cytosolic domain of IRE1 induces the cleavage of the X-box binding protein 1 (XBP1) mRNA by the splicing of a 26-nucleotide intron from human XBP1 mRNA, leading to the spliced isoform of XBP1 (XBP1s) [64,65]. XBP1s is a basic leucine zipper transcription factor, while the unspliced isoform of XBP1 is unable to activate genes, and is likely a negative regulator of the XBP1s transcriptional activity [64]. XBP1s controls the transcription of many targets like chaperones, foldases and components of the ERAD and is involved in the regulation of lipid biosynthesis, glucose metabolism, insulin signaling and DNA repair [66,67,68,69,70,71].

While IRE1 can degrade its own mRNA, it can also target other transcripts by a conserved mechanism called regulated IRE1-dependent decay (RIDD) in which IRE1 cleaves and inactivates transcripts by harbouring a CUGCAG sequence [72]. RIDD is involved in the maintenance of ER homeostasis by reducing the protein load by mRNA degradation [72,73], and it is proposed that the basal activity of RIDD is progressively increased with the severity of the ER stress. Interestingly, IRE1β was found to selectively induce translational repression through the 28S ribosomal RNA cleavage, demonstrating that IRE1α and IRE1β display different activities [59,74].

### 3.4. PERK Signaling

PERK is a ubiquitous serine/threonine kinase composed of an ER luminal domain and a cytoplasmic kinase domain [75]. To be activated, PERK has to be released from BiP, isoligomerized and trans-autophosphorylated [42,76]. Once activated, it phosphorylates eIF2α, which is a subunit of the eIF2 heterotrimer [75,76], itself regulating the initiation of protein synthesis by favoring the binding of the initiator tRNA to the 40S ribosomal subunits [77]. However, eIF2α phosphorylation inhibits eukaryotic translation initiation factor 2B and thereby, down-regulates protein synthesis, consequently reducing the protein load in the ER [78,79]. Remarkably, some transcripts are more efficiently translated when PERK inhibits the global translation in cells. The ubiquitously expressed activating transcription factor 4 (ATF4), whose transcript contains short upstream open reading frames (uORFs), is normally inefficiently translated [80,81]. However, attenuation of translation from uORFs shifts translation initiation towards the protein-coding AUG, resulting in more efficient synthesis of ATF4 [52]. ATF4 can then bind to the promoter of CAAT/enhancer-binding protein (C/EBP) homologous protein CHOP and induce its expression [52]. ATF4 and CHOP directly induce genes involved in protein synthesis and in UPR, but conditions under which they increase protein synthesis can result in ATP depletion, oxidative stress and cell death [82,83]. Phosphorylated eIF2α also directly enhances the translation of CHOP and other proteins involved in the ER stress response, such as GADD34 which indirectly dephosphorylates eIF2α [84]. This creates a negative feedback loop, restoring protein synthesis. The interplay between GADD34, ATF4 and CHOP results in apoptosis.

### 3.5. Noncoding RNAs and PIWI Proteins

Noncoding RNAs are linked to ATF6, IRE1 and PERK in physiological and pathological conditions [85,86]. Noncoding RNAs are mainly microRNAs (miRNAs) and long noncoding RNAs (lncRNAs). miRNAs regulate protein expression by translational repression and mRNA degradation, the latest being more common. They are involved in apoptosis, inflammation, hypoxia, oxidative stress and UPR. During UPR, mRNA levels are decreased by miRNAs. Their expression as for lncRNAs can be modulated by the sensors of the UPR or by their downstream components. Some miRNAs regulate IRE1, which in turn regulates miRNAs by RIDD. One miRNA regulating PERK and ATF6 is modulated by other miRNAs. Upstream, they also regulate the expression of BiP. lncRNAs exhibit a similar role regarding the regulation of UPR. Their expression varies with the cell stress and the pathophysiological context. Some small noncoding RNAs (ncRNAs) also play a role in UPR; this interplay between noncoding RNAs and the UPR makes the network complex and underlines the fine regulation of ER stress responses [87,88].

During the UPR, PIWI proteins contribute to apoptosis, at least in human airway epithelial cells [89,90].

## 4. Inflammation and UPR in CF

### 4.1. Inflammation

Inflammation is dedicated to alleviating harmful stimuli. Whereas acute inflammation is advantageous and focused on the injured site, chronic inflammation is an issue because it inflames the whole tissues, recruits immune cells, and becomes harmful [91]. In CF, the progressive lung destruction is mainly due to inflammation, which starts shortly after birth [92]. Chronic inflammation is characterized by the secretion of inflammatory mediators and by the infiltration by polymorphonuclear neutrophils (PMN) leading to an altered lung function in CF [93]. While inflammation can be triggered in the absence of infection, they are both tightly linked. TLRs recognize molecular patterns on pathogens and activate inflammatory cells that produce NF-κB, growth factors, chemokines and the pro-inflammatory cytokines IL-8 and TNF-α. IL-8 recruits PMN and TNF-α raises the expression of endothelial cell adhesion molecules in capillaries [94]. Whereas many inflammatory targets such as MMP-9, ICAM-1, VCAM-1, COX-2 and cPLA2 are implicated in inflammation, its main marker is NF-κB [95]; its translocation to the nucleus, where it increases the expression of pro-inflammatory genes, occurs when CFTR is altered or inhibited [96]. IL-8 secretion by the epithelial cells leads to the PMN invasion followed by the secretion of pro-inflammatory mediators such as TNF-α, IL-1β, IL-6, IL-17, IL-33, GM-CSF, G-CSF and HMGB1 [97]. One of the key cytokines governing inflammation in the CF lung is IL-1β. Together with TNFα, it enhances the PMN secretory responses and the expression of adhesion molecule on endothelial cells, promoting cachexia. Inflammation in CF is deregulated and is unable to complete; indeed, CF airways are deficient in regulatory molecules including IL-10, NO and lipoxin-A4 [97,98,99]. The role of the anti-inflammatory cytokine IL-10 is to complete the acute inflammation by decreasing the pro-inflammatory cytokines and by the induction of apoptosis in PMN. Hyper inflammation in CF is due to an altered balance between inflammatory and anti-inflammatory cytokines in which miRNAs are suspected to be involved, because miR-199a-3p negatively regulates the NF-κB pathway [99,100,101], while lipids also play a role. When the cholesterol homeostasis and its mistrafficking are restored, pro-inflammatory signaling is reduced [102]; indeed, macrophages, lymphocytes and airway smooth muscle cells are also implicated. Broncho alveolar lavage from CF children contains two subtypes of macrophages [103]: the first one is responsible for clearing the lung of microbes and for producing large amounts of TNF-α, IL-1β, IL-8; the second subtype is involved in the tissue repair by the release of IL-4, IL-13 and IL-10. Nevertheless, macrophages in CF lungs fail to eradicate bacteria [104,105].

The CFTR protein is itself involved in inflammation, and when it is defective in PMN, chlorination of pathogens is impaired and in T-cells less IL-10 is produced [106,107]. Wt-CFTR inhibits the production of IL-8, its loss of function induces the production of IL-8, NF-κB and cytokines [108,109].

### 4.2. UPR and Inflammation Are Functionally Linked

The UPR can either alleviate or impede inflammatory pathways. This is observed in many conditions, such as viral infection, overload of free cholesterol in macrophages and in acute lung injury (ALI). Indeed, the inhibition of BiP decreases the levels of inflammatory mediators and prevents the activation of NF-κB through ATF6 [110]. IRE1 is involved in the inflammation in mucous cells of the airways and in pancreatic beta cells where the IRE1/XBP1 pathway potentiates the activation of NFκB. The downregulation of XBP1 and the phosphorylation of IRE1α decrease the expression of inflammatory molecules, whereas the overexpression of XBP1 blocks the IRE1α/IKK/NF-κB pathway [111]. In some situations, IRE1α activates the transcription of cytokines through the degradation of IκB by IKK-mediated phosphorylation [112]. In a reverse mode, the NF-κB activation promotes ER stress and the production of TNF-α and maintains the inflammatory state [113]. Another example of the interplay between IRE1 and inflammation is the decreased IL-1β and IL-18 production when the RNase function of IRE1α is inhibited [114]. A last piece of evidence showing the crosstalk between UPR and inflammation is the activation of the NF-κB pathway through the phosphorylation of eIF2α [115]; some works showed that the UPR activates the p65/p50 NF-κB subunits, what is abolished when PERK is lacking. PERK increases the activation of NF-κB due to the suppression of synthesis of IκBα, and in addition, phosphorylated IRE1α can recruit TRAF2 to facilitate NF-κB activation [116].

In CF, UPR plays a role upon inflammation through an increased production of IL-8 and IL-6 [117]. Whereas, UPR activates inflammation by the classic NF-κB activation through an interaction between IRE1α and TRAF2 and subsequent activation of the IKK complex [118], it also plays a role through an unconventional NF-κB activation, independent of the IκBα phosphorylation [119,120]. Furthermore, XBP1 can lead to the production of IL-6 and TNF-α [121,122]. The events are not so simple because the activation of NF-κB by UPR depends on the ER stressors and on the cell type [123].

**Figure 2:**
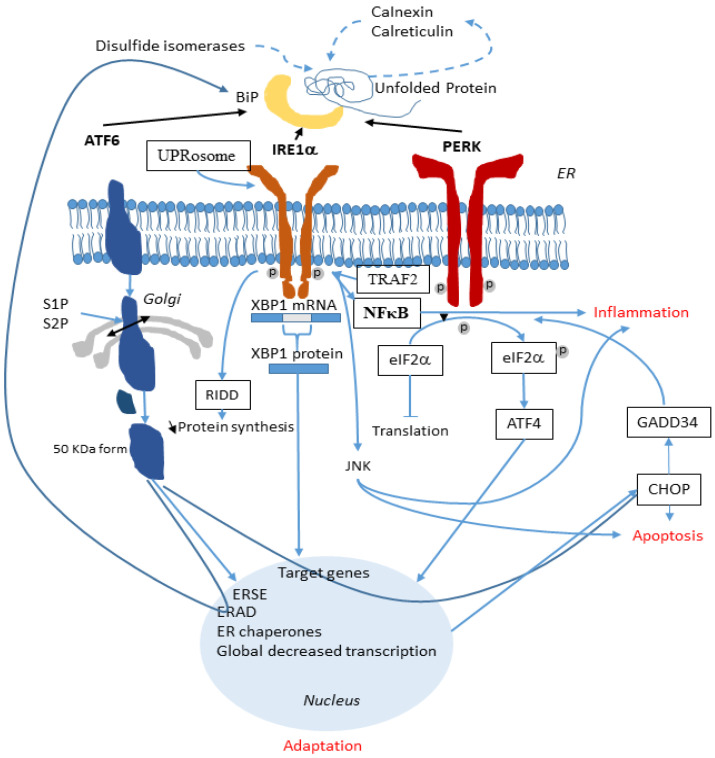
Involvement of the UPR in the inflammatory response. In the ER, when ATF6, IRE1α and PERK are released from BiP, they activate the UPR (black lines). ATF6 is cleaved by S1P and S2P and its active form migrates to the nucleus. IRE1α splices XBP1 mRNA and the XBP1 protein is translocated to the nucleus. PERK phosphorylates eIF2α which also reach the nucleus. These three arms of the UPR activate specific genes aimed at alleviating the ER stress. During this process, inflammation is triggered (blue lines). A synergistic effect of both IRE1α and PERK permits the complete NF-κB activation through an interaction between IRE1α and TRAF2 and through the JNK pathway. UPR also activates the release of the pro-inflammatory IL-6 and IL-8. NF-κB is the main link between UPR and inflammation.

ER stress-induced inflammation is central to the pathogenesis of numerous human intestinal, metabolic, and airway diseases including CF. UPR may suppress the secretion of IL-8 in CF airways or at the opposite, positively regulate its production, depending on the specific inflammatory stimulus and the cell type. A synergistic effect of both IRE1α and PERK is needed for a complete NF-κB activation [124]. A schematic representation of the interplay between UPR and the inflammatory response is shown in Figure 2.

## 5. Infection and UPR in CF

### 5.1. Infection in CF

Dysbiosis triggers the immune response and favors the progression of CF. The decreased mucociliary clearance allows pathogenic species to colonize niches due to an altered oxygen availability, temperature and pH. Together with an impaired innate immunity, it favors chronic infections and alters the pulmonary function. More than 1000 species were identified in CF airways, anaerobics being fewer than in normal lung [125]; 90% of CF patients are infected by mucoid *P. aeruginosa* that produces virulence factors such as extracellular toxins, proteases, haemolysins and alginate exopolysaccharides. The microbiome of patients becomes less diverse and is dominated by at least *the Burkholderia cepacian* complex (Bcc), *Stenotrophomonas maltophilia*, *Achromobacter xylosoxidans*, or *S. aureus*. *P. aeruginosa* and *B. cepacia*, while both modify the presence of other species and worsen the severity of the disease [126]. The p.Phe508del mutation is associated with *P. aeruginosa* [127]; it is observed in 25% of children and in 70% of adults. Members of the Bcc are responsible for the fatal ‘*cepacia* syndrome’ (4% of the patients) characterized by necrosis, bacteremia and sepsis [128]. *Haemophilus* *influenzae* is mainly found in patients (32%) between 2 to 5 years old. Microbiologists now consider the microbial environment as a whole, since the intestinal microbiome may modify the pulmonary one via metabolite exchange.

**Figure 3:**
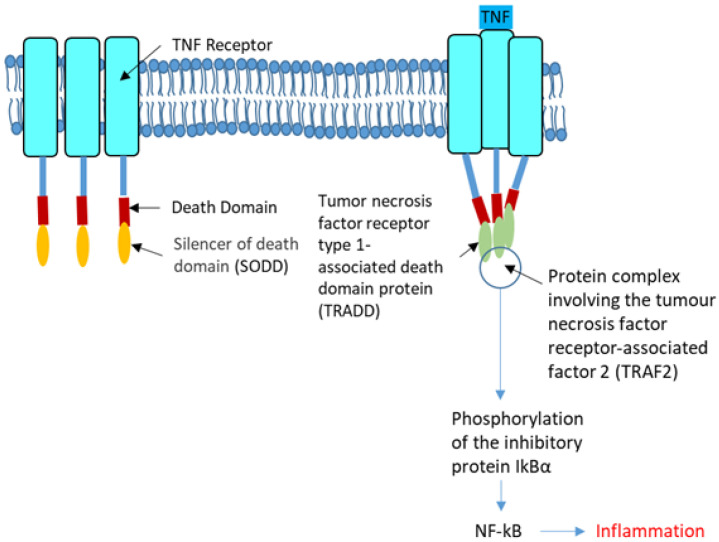
The tumor necrosis factor receptor. The tumor necrosis factor receptor (TNFR, left) is a cytokine receptor characterized by its ability to bind the tumor necrosis factors (TNFs). This binding occurs via an extracellular cysteine-rich domain. It has a death domain involved in the regulation of apoptosis and inflammation through their activation of caspases and NF-κB. In the strict sense, the term TNF receptor is used to refer to the archetypal members of the superfamily, namely TNFR1 which recognize TNF-α. Its SODD (silencer of death domains), suppresses TNF-induced cell death and NF-κB activation. Most of the TNF receptors need a specific adaptor protein such as TRADD for downstream signaling (Right). TRADD is an adaptor molecule that interacts with TNFR1 and mediates NF-κB activation. The tumor necrosis factor receptor-associated factor 2 (TRAF2) is required for TNF-alpha-mediated activation of MAPK8/JNK and NF-κB. The configuration shown in the right panel permits the phosphorylation of the inhibitory protein IkBα that favors the activation of NF-κB.

In CF, airway cells release high amounts of IL-8 in the absence of bacterial infection [129]. During infection, the immune system recognizes microbes through microbial-associated molecular patterns (MAMPs) to specific pattern recognition receptors (PRRs: TLRs, NLRs, RLRs and ALRs) interactions [130]. NOD-like receptors (NLRs) contain NOD1 and NOD2 that are involved in the recognition of peptidoglycan moieties from bacteria. In addition to NF-κB, NOD1 and NOD2 can activate the p38, ERK, and MAPKs [131]. NODs can be induced by TLR, IFN-γ and TNF-α. High levels of TNFα worsen CF due to its action upon NF-κB, P65 and the downstream release of IL-6 and IL-8 into the airways [132]; it induces the phosphorylation of IkBα allowing NF-κB to stimulate the transcription of cytokines like IL-8. Whereas Wt-CFTR binds to the tumor necrosis factor receptor type 1-associated DEATH domain protein (TRADD), it is not the case for p.F508del-CFTR. NF-κB and TNFα activation are higher in p.F508del-CFTR cells than in Wt-CFTR cells, but this difference is abolished when TRADD is knocked down (Figure 3).

**Figure 4:**
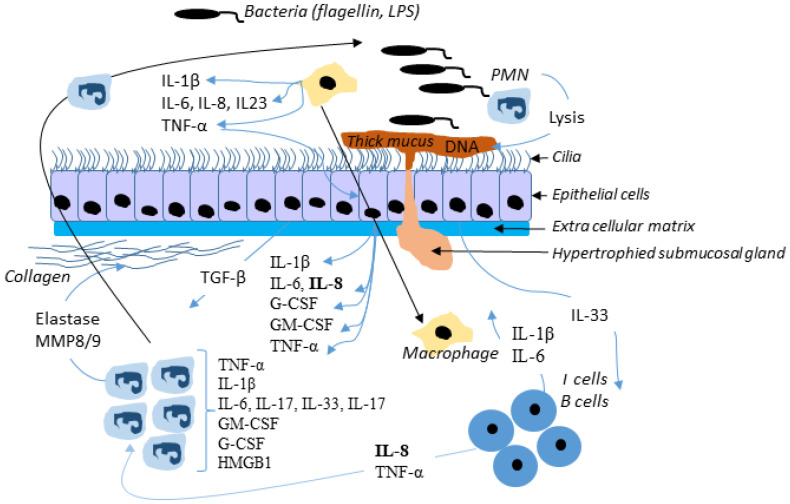
Simplified view of infection in the CF airway. In response to bacterial invasion, neutrophils migrate from submucosal regions through airway epithelial cells to reach airway lumen, where they encounter with pathogens and secret inflammatory mediators and release proteases and oxidants. This damage the airway and chemoattractants stimulate further neutrophil influx. Although the neutrophil and epithelial cell and their mediators have been most intensely studied, many other cells, including dendritic cells, T-lymphocytes, B-lymphocytes, macrophages, and airway smooth muscle cells, produce inflammatory mediators and are actively involved in the host inflammatory response in CF. Not all mediators and cell types implicated in CF are shown here.

Therefore, Wt-CFTR controls TRADD and modulates the activation of NF-κB by TNFα which increases the cell surface expression and function of F508del-CFTR, in human bronchial epithelial cells [109]. The connection between infection and inflammation is thus obvious. Bacteria release LPS, which acts through TLRs to activate NFκB signaling; however, NFκB-activated inflammation persists when infection is suppressed in CF airways [133]. Figure 4 shows a simplified view of infection in the CF airway.

### 5.2. UPR and Infection Are Functionally Linked

Infection is an ER stressor; the UPR senses pathogenic danger and transforms the stress signal into immune response [134,135], and is also involved in the activation of B cells, in the survival of dendritic cells, in the differentiation of eosinophils and shapes the immune responses in many other cell types. The protective effect of the UPR during protein synthesis due to infection and its involvement in the maturation of immune cells are other points showing its link with infection. The UPR influences the response of cells to pathogens by stimulating the recognition of receptors and by regulating the transcription factors of cytokines. Among the nuclear hormone receptor superfamily, PPARγ is involved in the regulation of insulin response, cell proliferation, lipid metabolism and inflammation [134]. Its decreased expression in the CF airway epithelia is due to the induction of CHOP [136]. Therefore, UPR is likely involved in the PPARγ mediated inflammation due to bacteria, via the PERK pathway.

UPR also tunes the modulation of interleukins, interferons and the TNF family [137]. In sterile UPR triggering, cells are more sensitive to PRR stimulation, leading to an increased production of IL-6, TNF-α, IL-23, and IFN-β. For IL-6, TNF-α and IFN-β, synergy between ER stress and PRR ligation is XBP1-dependent [138]. For IL-23, it is CHOP-dependent. ER stress may enable cells to produce IL-1β in response to TLR4 ligation, but in a XBP1 and CHOP independent manner [139]. Conversely, PRRs activation partially activates some UPR pathways and selectively suppresses others. TLR3 or TLR4 stimulation suppresses the activation of ATF4 and CHOP, while stimulation of TLR2 and TLR4 activates IRE1 that induces the XBP1 mRNA splicing and the binding of XBP1 to cytokine promoters, without folding chaperon’s synthesis. TLR signaling does not trigger PERK and ATF6 pathways [140]. Another example of partial activation of UPR is seen in viral infections in which the release of dsRNA stimulates eIF2α phosphorylation and GADD34 induction [141]. The basis of this specificity remains unclear; indeed, why TLR4 induced XBP1 and promotes the production of cytokines without enhancing the production of its own chaperone targets is still not understood. The partial UPR signaling and modulation in response to PRR activation has been termed as the “microbial stress response” pathway [142]. Interestingly, the UPR adaptation to PRR activation permits enhancing of the production of cytokines without the risk of the triggering of apoptosis, as if UPR was fully engaged. The type of ER stress is also important for the function of immune cells and for the UPR-cytokine crosstalk.

**Figure 5:**
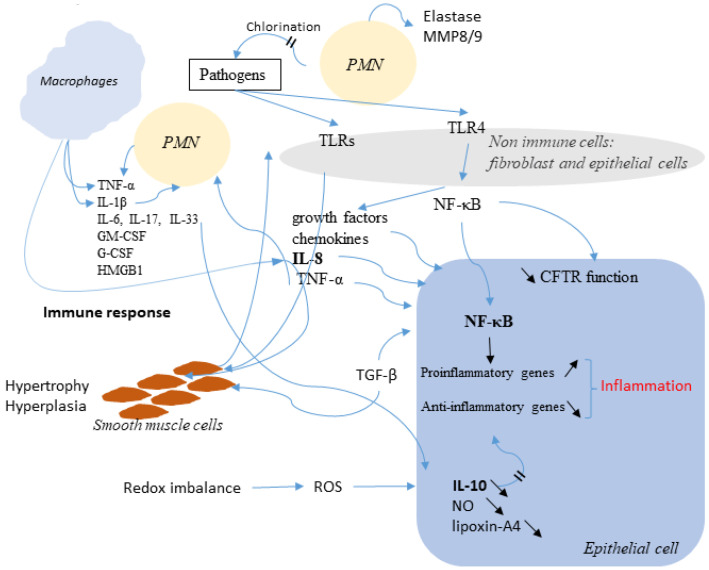
Schematic representation of the link between infection, inflammation, and CFTR. Immune cells release many factors, such as TNF-α, IL-1β, IL-8, IL-4, IL-13 and IL-10 in order to eradicate pathogens. Among the secreted molecules, some are pro-inflammatory like Il-8 and TNF-α. NF-κB is activated. The altered function of CFTR is also involved and is accompanied by an increased expression of NF-κB which activates inflammatory genes. The Redox imbalance also takes part in the activation of the inflammatory response.

UPR is induced in response to bacterial pathogens, resulting in enhanced pro-inflammatory cytokine induction. This bacterial induced UPR occurs through either bacterial products or intracellular lifecycles and the UPR modulation of host immunity. *Escherichia coli* produces a subtilase toxin that cleaves BiP, triggering the three arms of the UPR [138], and this UPR activation promotes apoptosis or attenuates NF-κB responses. Besides the secretion of factors, some bacteria form spatial relationships with the ER during their intracellular lifecycle. For instance, *Legionella* and *Brucella* traffic in the endosomal pathway and establish replicative vacuoles in compartments derived from the ER [143], while *Legionella* inhibits XBP1 splicing. The activation of IRE1 due to TLR signaling induces a strong pro-inflammatory cytokine induction. Nevertheless, *Legionella pneumophila*, which is an intravacuolar pathogen that replicates in an ER-associated compartment, inactivates IRE1 despite an active TLR signaling [144]; indeed, *Legionella* *pneumophila* can inhibit chemical and bacterial induction of XBP1 splicing. *A* microtubule stabilizing factor with UPR-inducing properties is secreted by *Brucella* [145]; it is responsible for restructuration of the ER, which becomes condensed and fragmented. Whereas Chlamydia infection induces transient BiP upregulation and eIF2α phosphorylation but no ATF6 cleavage, it triggers IRE1 activation and XBP1 splicing and induces CHOP [146,147]. UPR enables infected cells to sense invasion by pathogens and during infection, providing greater cytokine responses when threats impact cell function than when there is only a PRRs activation. The balance between the PRR stimulation and the degree of ER stress sways the cell to either UPR or microbial stress response. Figure 5 is a schematic representation of the link between infection, inflammation, and CFTR.

## 6. Discussion and Conclusions

**Figure 6:**
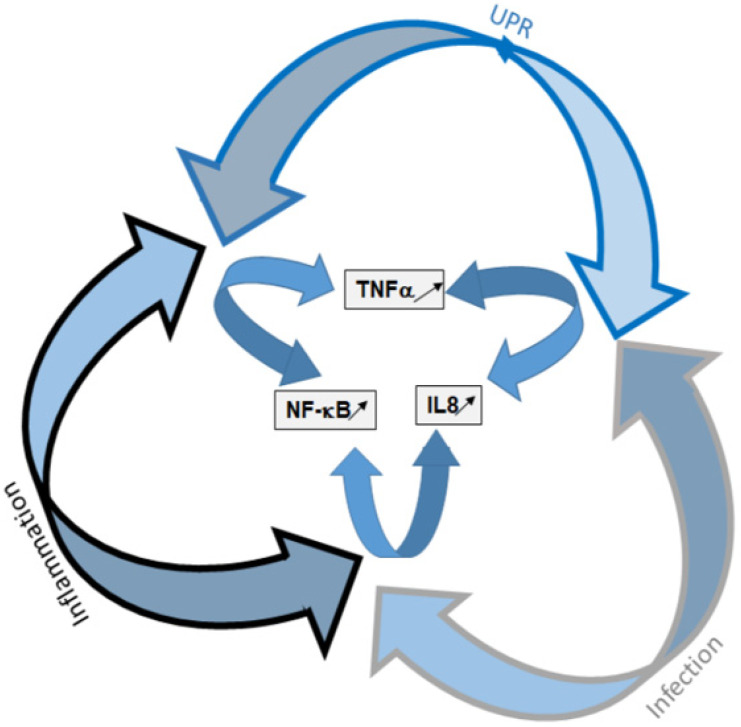
Schematic representation of the UPR, infection and inflammation interplay. UPR and its components are presented in blue, infection is in gray and inflammation in black. The three arms of the UPR are involved in the triggering of inflammation and infection. The three pathways share common elements that are mainly NF-κB, TNF-α and IL-8.

Misfolded CFTR proteins that are produced in CF are retained within the ER and rapidly degraded by the ERAD. This is in particular the case for the most frequent mutation, p.Phe508del. This retention within the ER can induce an ER stress, leading to the triggering of UPR that is a physiological cellular recovery process aimed to regulate the protein load of the ER. Whether the UPR is triggered due to the retention of p.Phe508del-CFTR itself and/or to a protein overload of the ER, is still in debate. Nevertheless, some line of evidence suggests that it is triggered, at least in an unconventional way. In our opinion, there is likely a threshold above which the proteins retained in the ER trigger the UPR and, as in other diseases due to unfolding, the threshold is almost reached in CF. The UPR is responsible for the activation of degradation genes of the ERAD, the increased expression of chaperons and limits the global protein synthesis in cells. It limits the expression of the p.Phe508del-CFTR itself, by the activation of ATF6. Therefore, the hypothesis that it is likely triggered but becomes obvious when other events happen, including infection and/or inflammation, that also contribute the UPR triggering. Whatever its triggers, the UPR is present in CF and a fine regulation of the balance between infection, inflammation and UPR occurs. When at least one of these three components is triggered, the two others likely evolve towards their activation. Indeed, we show here that many pathways are shared by the three processes, showing an interplay between them (Figure 6). Among the shared elements are TNFα, IL8 and NF-κB which are well known to participate to the pathophysiology in CF.

In conclusion, because infection, inflammation and UPR are indissociable, we propose that UPR deserves to be considered as equal as the other two processes. Indeed, even if it is atypical, UPR activation in CF sensitizes the innate immune system and is involved in the inflammatory response. Strategies aimed to modulate the UPR pathways may be a novel therapeutic approach to alleviate p.Phe508del-CFTR defects.

## Abbreviations

ATF6/4	activating transcription factor 6/4
BiP	Binding Immunoglobulin protein
CHOP	CAAT/enhancer-binding protein (C/EBP) homologous protein
COX-2	cyclooxygenase-2
cPLA2	cytosolic phospholipase A2
CREB	cAMP response element-binding protein
CREB3L3	cAMP responsive element-binding protein 3 like 3
eIF2α	eukaryotic translation initiation factor 2α
ERAD	Endoplasmic-reticulum-associated protein degradation
ERQC	ER Quality Control
ERSE	ER stress response element
GADD34	growth arrest and DNA-damage-inducible 34
GM-CSF	Granulocyte-macrophage colony-stimulating factor
GRP78	glucose-regulated protein 78
GSK-3β	Glycogen synthase kinase 3 beta
HMGB1	High-mobility group box 1
ICAM-1	intercellular adhesion molecule-1
IKK	IκB kinase
IRE1	inositol-requiring enzyme 1
IκB	inhibitor of nuclear factor kappa B
LUMAN	cAMP responsive element-binding protein 3 or CREB3
MAMPs	microbial-associated molecular patterns
MMP-9	matrix metalloproteinase-9
NF-κB	nuclear factor kappa-light-chain-enhancer of activated B cells
NLRs	NOD-like receptors
NOD1/2	Nucleotide-binding oligomerization domain-containing protein 1/2
PERK	protein kinase RNA (PKR)-like ER kinase
PPARγ	Peroxisome proliferator-activated receptor γ
PRRs	pattern recognition receptors
RIDD	IRE1-dependent decay
TLR3/TLR4	Toll-like receptors 3/4
TRADD	Tumor necrosis factor receptor type 1-associated DEATH domain protein
TRAF2	TNFα receptor-associated factor 2
TRAF2	tumor necrosis factor receptor-associated factor 2
VCAM-1	vascular cell adhesion molecule-1
XBP1	X-box binding protein 1

## References

[1] Riordan J.R., Rommens J.M., Kerem B., Alon N., Rozmahel R., Grzelczak Z., Zielenski J., Lok S., Plavsic N., Chou J.L. (1989). Identification of the cystic fibrosis gene: Cloning and characterization of complementary DNA. Science.

[2] Rich D.P., Anderson M.P., Gregory R.J., Cheng S.H., Paul S., Jefferson D.M., McCann J.D., Klinger K.W., Smith A.E., Welsh M.J. (1990). Expression of cystic fibrosis transmembrane conductance regulator corrects defective chloride channel regulation in cystic fibrosis airway epithelial cells. Nature.

[3] Anderson M.P., Gregory R.J., Thompson S., Souza D.W., Paul S., Mulligan R.C., Smith A.E., Welsh M.J. (1991). Demonstration that CFTR is a chloride channel by alteration of its anion selectivity. Science.

[4] Berdiev B.K., Qadri Y.J., Benos D.J. (2009). Assessment of the CFTR and ENaC association. Mol. Biosyst..

[5] Heeckeren A., Walenga R., Konstan M.W., Bonfield T., Davis P.B., Ferkol T. (1997). Excessive inflammatory response of cystic fibrosis mice to bronchopulmonary infection with *Pseudomonas Aeruginosa*. J. Clin. Investig..

[6] Pier G.B., Grout M., Zaidi T.S., Olsen J.C., Johnson L.G., Yankaskas J.R., Goldberg J.B. (1996). Role of mutant CFTR in hypersusceptibility of cystic fibrosis patients to lung infections. Science.

[7] Cheng S.H., Gregory R.J., Marshall J., Paul S., Souza D.W., White G.A., O’Riordan C.R., Smith A.E. (1990). Defective intracellular transport and processing of CFTR is the molecular basis of most cystic fibrosis. Cell.

[8] Liu C.Y., Kaufman R.J. (2003). The unfolded protein response. J. Cell Sci..

[9] Kaufman R.J. (1999). Stress signaling from the lumen of the endoplasmic reticulum: Coordination of gene transcriptional and translational controls. Genes Dev..

[10] Schröder M., Kaufman R.J. (2005). The mammalian unfolded protein response. Annu. Rev. Biochem..

[11] Ribeiro C.M.P., Boucher R.C. (2010). Role of endoplasmic reticulum stress in cystic fibrosis-related airway inflammatory responses. Proc. Am. Thorac. Soc..

[12] Van’t Wout E.F.A., van Schadewijk A., van Boxtel R., Dalton L.E., Clarke H.J., Tommassen J., Marciniak S.J., Hiemstra P.S. (2015). Virulence factors of *Pseudomonas Aeruginosa* induce both the unfolded protein and integrated stress responses in airway epithelial cells. PLoS Pathog..

[13] Nanua S., Sajjan U., Keshavjee S., Hershenson M.B. (2006). Absence of typical unfolded protein response in primary cultured cystic fibrosis airway epithelial cells. Biochem. Biophys. Res. Commun..

[14] Kerbiriou M., Le Drévo M.-A., Férec C., Trouvé P. (2007). Coupling cystic fibrosis to endoplasmic reticulum stress: Differential role of Grp78 and ATF6. Biochim. Biophys. Acta.

[15] Rab A., Bartoszewski R., Jurkuvenaite A., Wakefield J., Collawn J.F., Bebok Z. (2007). Endoplasmic reticulum stress and the unfolded protein response regulate genomic cystic fibrosis transmembrane conductance regulator expression. Am. J. Physiol. Cell Physiol..

[16] Bartoszewski R., Rab A., Jurkuvenaite A., Mazur M., Wakefield J., Collawn J.F., Bebok Z. (2008). Activation of the unfolded protein response by DeltaF508 CFTR. Am. J. Respir. Cell Mol. Biol..

[17] Hull-Ryde E.A., Minges J.T., Martino M.E.B., Kato T., Norris-Drouin J.L., Ribeiro C.M.P. (2021). IRE1α is a therapeutic target for cystic fibrosis airway inflammation. Int. J. Mol. Sci..

[18] Ribeiro C.M.P., Lubamba B.A. (2017). Role of IRE1α/XBP-1 in cystic fibrosis airway inflammation. Int. J. Mol. Sci..

[19] Bakunts A., Orsi A., Vitale M., Cattaneo A., Lari F., Tadè L., Sitia R., Raimondi A., Bachi A., van Anken E. (2017). Ratiometric sensing of BiP-client versus BiP levels by the unfolded protein response determines its signaling amplitude. eLife.

[20] Vitale M., Bakunts A., Orsi A., Lari F., Tadè L., Danieli A., Rato C., Valetti C., Sitia R., Raimondi A. (2019). Inadequate BiP availability defines endoplasmic reticulum stress. eLife.

[21] Blohmke C.J., Mayer M.L., Tang A.C., Hirschfeld A.F., Fjell C.D., Sze M.A., Falsafi R., Wang S., Hsu K., Chilvers M.A. (2012). Atypical activation of the unfolded protein response in cystic fibrosis airway cells contributes to P38 MAPK-mediated innate immune responses. J. Immunol..

[22] Dobson C.M. (2004). Principles of protein folding, misfolding and aggregation. Semin. Cell Dev. Biol..

[23] Karplus M., Weaver D.L. (1976). Protein-folding dynamics. Nature.

[24] Ohgushi M., Wada A. (1983). “Molten-Globule State”: A compact form of globular proteins with mobile side-chains. FEBS Lett..

[25] Thomas P.J., Ko Y.H., Pedersen P.L. (1992). Altered protein folding may be the molecular basis of most cases of cystic fibrosis. FEBS Lett..

[26] Bychkova V.E., Ptitsyn O.B. (1995). Folding intermediates are involved in genetic diseases?. FEBS Lett..

[27] Drumm M.L., Wilkinson D.J., Smit L.S., Worrell R.T., Strong T.V., Frizzell R.A., Dawson D.C., Collins F.S. (1991). Chloride conductance expressed by Delta F508 and other mutant CFTRs in *Xenopus Oocytes*. Science.

[28] Dalemans W., Barbry P., Champigny G., Jallat S., Dott K., Dreyer D., Crystal R.G., Pavirani A., Lecocq J.P., Lazdunski M. (1991). Altered chloride ion channel kinetics associated with the Delta F508 cystic fibrosis mutation. Nature.

[29] Choi M.Y., Partridge A.W., Daniels C., Du K., Lukacs G.L., Deber C.M. (2005). Destabilization of the transmembrane domain induces misfolding in a phenotypic mutant of cystic fibrosis transmembrane conductance regulator. J. Biol. Chem..

[30] Ward C.L., Kopito R.R. (1994). Intracellular turnover of cystic fibrosis transmembrane conductance regulator. Inefficient processing and rapid degradation of wild-type and mutant proteins. J. Biol. Chem..

[31] Gottesman S., Wickner S., Maurizi M.R. (1997). Protein quality control: Triage by chaperones and proteases. Genes Dev..

[32] Pind S., Riordan J.R., Williams D.B. (1994). Participation of the endoplasmic reticulum chaperone calnexin (P88, IP90) in the biogenesis of the cystic fibrosis transmembrane conductance regulator. J. Biol. Chem..

[33] Harada K., Okiyoneda T., Hashimoto Y., Ueno K., Nakamura K., Yamahira K., Sugahara T., Shuto T., Wada I., Suico M.A. (2006). Calreticulin negatively regulates the cell surface expression of cystic fibrosis transmembrane conductance regulator. J. Biol. Chem..

[34] Knittler M.R., Dirks S., Haas I.G. (1995). Molecular chaperones involved in protein degradation in the endoplasmic reticulum: Quantitative interaction of the heat shock cognate protein BiP with partially folded immunoglobulin light chains that are degraded in the endoplasmic reticulum. Proc. Natl. Acad. Sci. USA.

[35] McCracken A.A., Brodsky J.L. (2005). Recognition and delivery of ERAD substrates to the proteasome and alternative paths for cell survival. Curr. Top. Microbiol. Immunol..

[36] El Khouri E., Le Pavec G., Toledano M.B., Delaunay-Moisan A. (2013). RNF185 is a novel E3 ligase of endoplasmic reticulum-associated degradation (ERAD) that targets cystic fibrosis transmembrane conductance regulator (CFTR). J. Biol. Chem..

[37] Rubenstein R.C., Zeitlin P.L. Sodium 4-phenylbutyrate Downregulates Hsc70: Implications for Intracellular Trafficking of DeltaF508-CFTR. https://pubmed.ncbi.nlm.nih.gov/10666020/.

[38] Meacham G.C., Lu Z., King S., Sorscher E., Tousson A., Cyr D.M. (1999). The Hdj-2/Hsc70 Chaperone pair facilitates early steps in CFTR biogenesis. EMBO J..

[39] Zhang Y., Nijbroek G., Sullivan M.L., McCracken A.A., Watkins S.C., Michaelis S., Brodsky J.L. (2001). Hsp70 molecular Chaperone facilitates endoplasmic reticulum-associated protein degradation of cystic fibrosis transmembrane conductance regulator in yeast. Mol. Biol. Cell.

[40] Fu L., Rab A., Tang L.P., Bebok Z., Rowe S.M., Bartoszewski R., Collawn J.F. (2015). ΔF508 CFTR surface stability is regulated by DAB2 and CHIP-mediated ubiquitination in post-endocytic compartments. PLoS ONE.

[41] Ron D., Walter P. (2007). Signal integration in the endoplasmic reticulum unfolded protein response. Nat. Rev. Mol. Cell Biol..

[42] Bertolotti A., Zhang Y., Hendershot L.M., Harding H.P., Ron D. (2000). Dynamic interaction of BiP and ER stress transducers in the unfolded-protein response. Nat. Cell Biol..

[43] Gething M.J., Sambrook J. (1992). Protein folding in the cell. Nature.

[44] Pobre K.F.R., Poet G.J., Hendershot L.M. (2019). The endoplasmic reticulum (ER) Chaperone BiP is a master regulator of ER functions: Getting by with a little help from ERdj friends. J. Biol. Chem..

[45] Ting J., Lee A.S. (1988). Human Gene Encoding the 78,000-Dalton Glucose-Regulated Protein and Its Pseudogene: Structure, Conservation, and Regulation.

[46] Melnyk A., Rieger H., Zimmermann R. (2015). Co-Chaperones of the mammalian endoplasmic reticulum. Subcell. Biochem..

[47] Preissler S., Rato C., Chen R., Antrobus R., Ding S., Fearnley I.M., Ron D. (2015). AMPylation matches BiP activity to client protein load in the endoplasmic reticulum. eLife.

[48] Gopal U., Pizzo S.V. (2021). Cell surface GRP78 signaling: An emerging role as a transcriptional modulator in cancer. J. Cell. Physiol..

[49] Okada T., Haze K., Nadanaka S., Yoshida H., Seidah N.G., Hirano Y., Sato R., Negishi M., Mori K. A Serine Protease Inhibitor Prevents Endoplasmic Reticulum Stress-Induced Cleavage but Not Transport of the Membrane-Bound Transcription Factor ATF6. https://pubmed.ncbi.nlm.nih.gov/12782636/.

[50] Shen J., Chen X., Hendershot L., Prywes R. (2002). ER stress regulation of ATF6 localization by dissociation of BiP/GRP78 binding and unmasking of golgi localization signals. Dev. Cell.

[51] Ye J., Rawson R.B., Komuro R., Chen X., Davé U.P., Prywes R., Brown M.S., Goldstein J.L. (2000). ER stress induces cleavage of membrane-bound ATF6 by the same proteases that process SREBPs. Mol. Cell.

[52] Hai T.W., Liu F., Coukos W.J., Green M.R. (1989). Transcription factor ATF CDNA clones: An extensive family of leucine zipper proteins able to selectively form DNA-binding heterodimers. Genes Dev..

[53] Yang H., Niemeijer M., van de Water B., Beltman J.B. (2020). ATF6 is a critical determinant of CHOP dynamics during the unfolded protein response. iScience.

[54] Roy B., Lee A.S. (1999). The mammalian endoplasmic reticulum stress response element consists of an evolutionarily conserved tripartite structure and interacts with a novel stress-inducible complex. Nucleic Acids Res..

[55] Yoshida H., Haze K., Yanagi H., Yura T., Mori K. (1998). Identification of the Cis-acting endoplasmic reticulum stress response element responsible for transcriptional induction of mammalian glucose-regulated proteins. Involvement of basic leucine zipper transcription factors. J. Biol. Chem..

[56] Haze K., Okada T., Yoshida H., Yanagi H., Yura T., Negishi M., Mori K. (2001). Identification of the G13 (CAMP-response-element-binding protein-related protein) gene product related to activating transcription factor 6 as a transcriptional activator of the mammalian unfolded protein response. Biochem. J..

[57] Thuerauf D.J., Marcinko M., Belmont P.J., Glembotski C.C. (2007). Effects of the isoform-specific characteristics of ATF6 alpha and ATF6 beta on endoplasmic reticulum stress response gene expression and cell viability. J. Biol. Chem..

[58] Asada R., Kanemoto S., Kondo S., Saito A., Imaizumi K. (2011). The signalling from endoplasmic reticulum-resident BZIP transcription factors involved in diverse cellular physiology. J. Biochem..

[59] Iwawaki T., Hosoda A., Okuda T., Kamigori Y., Nomura-Furuwatari C., Kimata Y., Tsuru A., Kohno K. (2001). Translational control by the ER transmembrane kinase/ribonuclease IRE1 under ER stress. Nat. Cell Biol..

[60] Martino M.B., Jones L., Brighton B., Ehre C., Abdulah L., Davis C.W., Ron D., O’Neal W.K., Ribeiro C.M.P. (2013). The ER stress transducer IRE1β is required for airway epithelial mucin production. Mucosal Immunol..

[61] Ali M.M.U., Bagratuni T., Davenport E.L., Nowak P.R., Silva-Santisteban M.C., Hardcastle A., McAndrews C., Rowlands M.G., Morgan G.J., Aherne W. (2011). Structure of the Ire1 autophosphorylation complex and implications for the unfolded protein response. EMBO J..

[62] Sanches M., Duffy N.M., Talukdar M., Thevakumaran N., Chiovitti D., Canny M.D., Lee K., Kurinov I., Uehling D., Al-awar R. (2014). Structure and mechanism of action of the hydroxy-aryl-aldehyde class of IRE1 endoribonuclease inhibitors. Nat. Commun..

[63] Urano F., Wang X., Bertolotti A., Zhang Y., Chung P., Harding H.P., Ron D. (2000). Coupling of stress in the ER to activation of JNK protein kinases by transmembrane protein kinase IRE1. Science.

[64] Sidrauski C., Walter P. (1997). The transmembrane kinase Ire1p is a site-specific endonuclease that initiates MRNA splicing in the unfolded protein response. Cell.

[65] Yoshida H., Matsui T., Yamamoto A., Okada T., Mori K. (2001). XBP1 MRNA is induced by ATF6 and spliced by IRE1 in response to ER stress to produce a highly active transcription factor. Cell.

[66] Travers K.J., Patil C.K., Wodicka L., Lockhart D.J., Weissman J.S., Walter P. (2000). Functional and genomic analyses reveal an essential coordination between the unfolded protein response and ER-associated degradation. Cell.

[67] Iwakoshi N.N., Lee A.-H., Glimcher L.H. (2003). The X-box binding protein-1 transcription factor is required for plasma cell differentiation and the unfolded protein response. Immunol. Rev..

[68] Sriburi R., Jackowski S., Mori K., Brewer J.W. (2004). XBP1: A link between the unfolded protein response, lipid biosynthesis, and biogenesis of the endoplasmic reticulum. J. Cell Biol..

[69] So J.-S., Hur K.Y., Tarrio M., Ruda V., Frank-Kamenetsky M., Fitzgerald K., Koteliansky V., Lichtman A.H., Iwawaki T., Glimcher L.H. (2012). Silencing of lipid metabolism genes through IRE1α-mediated MRNA decay lowers plasma lipids in mice. Cell Metab..

[70] Zhou Y., Lee J., Reno C.M., Sun C., Park S.W., Chung J., Lee J., Fisher S.J., White M.F., Biddinger S.B. (2011). Regulation of Glucose Homeostasis through a XBP-1-FoxO1 Interaction. Nat. Med..

[71] Tao R., Chen H., Gao C., Xue P., Yang F., Han J.-D.J., Zhou B., Chen Y.-G. (2011). Xbp1-mediated histone H4 deacetylation contributes to DNA double-strand break repair in yeast. Cell Res..

[72] Tirasophon W., Lee K., Callaghan B., Welihinda A., Kaufman R.J. (2000). The endoribonuclease activity of mammalian IRE1 autoregulates its MRNA and is required for the unfolded protein response. Genes Dev..

[73] Maurel M., Chevet E., Tavernier J., Gerlo S. (2014). Getting RIDD of RNA: IRE1 in cell fate regulation. Trends Biochem. Sci..

[74] Imagawa Y., Hosoda A., Sasaka S.-I., Tsuru A., Kohno K. (2008). RNase domains determine the functional difference between IRE1alpha and IRE1beta. FEBS Lett..

[75] Shi Y., Vattem K.M., Sood R., An J., Liang J., Stramm L., Wek R.C. (1998). Identification and characterization of pancreatic eukaryotic initiation factor 2 alpha-subunit kinase, PEK, involved in translational control. Mol. Cell. Biol..

[76] McQuiston A., Diehl J.A. (2017). Recent insights into PERK-dependent signaling from the stressed endoplasmic reticulum. F1000Research.

[77] Lloyd M.A., Osborne J.C., Safer B., Powell G.M., Merrick W.C. (1980). Characteristics of eukaryotic initiation factor 2 and its subunits. J. Biol. Chem..

[78] Ernst H., Duncan R.F., Hershey J.W. (1987). Cloning and sequencing of complementary DNAs encoding the alpha-subunit of translational initiation factor EIF-2. characterization of the protein and its messenger RNA. J. Biol. Chem..

[79] Adams S.L., Safer B., Anderson W.F., Merrick W.C. (1975). Eukaryotic initiation complex formation. Evidence for two distinct pathways. J. Biol. Chem..

[80] Rowlands A.G., Panniers R., Henshaw E.C. (1988). The catalytic mechanism of guanine nucleotide exchange factor action and competitive inhibition by phosphorylated eukaryotic initiation factor 2. J. Biol. Chem..

[81] Harding H.P., Zhang Y., Bertolotti A., Zeng H., Ron D. (2000). Perk is essential for translational regulation and cell survival during the unfolded protein response. Mol. Cell.

[82] Harding H.P., Novoa I., Zhang Y., Zeng H., Wek R., Schapira M., Ron D. (2000). Regulated translation initiation controls stress-induced gene expression in mammalian cells. Mol. Cell.

[83] Vallejo M., Ron D., Miller C.P., Habener J.F. (1993). C/ATF, a member of the activating transcription factor family of DNA-binding proteins, dimerizes with CAAT/enhancer-binding proteins and directs their binding to CAMP response elements. Proc. Natl. Acad. Sci. USA.

[84] Palam L.R., Baird T.D., Wek R.C. (2011). Phosphorylation of EIF2 facilitates ribosomal bypass of an inhibitory upstream ORF to enhance CHOP translation. J. Biol. Chem..

[85] McMahon M., Samali A., Chevet E. (2017). Regulation of the unfolded protein response by noncoding RNA. Am. J. Physiol. Cell Physiol..

[86] Bartoszewska S., Kochan K., Madanecki P., Piotrowski A., Ochocka R., Collawn J.F., Bartoszewski R. (2013). Regulation of the unfolded protein response by MicroRNAs. Cell. Mol. Biol. Lett..

[87] Bisognin A., Sales G., Coppe A., Bortoluzzi S., Romualdi C. (2012). MAGIA^2^: From MiRNA and genes expression data integrative analysis to MicroRNA-transcription factor mixed regulatory circuits (2012 Update). Nucleic Acids Res..

[88] Bartoszewski R., Brewer J.W., Rab A., Crossman D.K., Bartoszewska S., Kapoor N., Fuller C., Collawn J.F., Bebok Z. (2011). The unfolded protein response (UPR)-activated transcription factor X-box-binding protein 1 (XBP1) induces MicroRNA-346 expression that targets the human antigen peptide transporter 1 (TAP1) MRNA and governs immune regulatory genes. J. Biol. Chem..

[89] Gebert M., Bartoszewska S., Janaszak-Jasiecka A., Moszyńska A., Cabaj A., Króliczewski J., Madanecki P., Ochocka R.J., Crossman D.K., Collawn J.F. (2018). PIWI proteins contribute to apoptosis during the UPR in human airway epithelial cells. Sci. Rep..

[90] Iwasaki Y.W., Siomi M.C., Siomi H. (2015). PIWI-Interacting RNA: Its biogenesis and functions. Annu. Rev. Biochem..

[91] Feghali C.A., Wright T.M. (1997). Cytokines in acute and chronic inflammation. Front. Biosci. J. Virtual Libr..

[92] Pillarisetti N., Williamson E., Linnane B., Skoric B., Robertson C.F., Robinson P., Massie J., Hall G.L., Sly P., Stick S. (2011). Infection, inflammation, and lung function decline in infants with cystic fibrosis. Am. J. Respir. Crit. Care Med..

[93] Ranganathan S.C., Parsons F., Gangell C., Brennan S., Stick S.M., Sly P.D., Australian Respiratory Early Surveillance Team for Cystic Fibrosis (2011). Evolution of pulmonary inflammation and nutritional status in infants and young children with cystic fibrosis. Thorax.

[94] Grommes J., Soehnlein O. (2011). Contribution of neutrophils to acute lung injury. Mol. Med. Camb. Mass.

[95] Lee I.-T., Yang C.-M. (2013). Inflammatory signalings involved in airway and pulmonary diseases. Mediat. Inflamm..

[96] Aghasafari P., George U., Pidaparti R. (2019). A Review of inflammatory mechanism in airway diseases. Inflamm. Res. Off. J. Eur. Histamine Res. Soc. AI.

[97] Bonfield T.L., Panuska J.R., Konstan M.W., Hilliard K.A., Hilliard J.B., Ghnaim H., Berger M. (1995). Inflammatory cytokines in cystic fibrosis lungs. Am. J. Respir. Crit. Care Med..

[98] Bonfield T.L., Konstan M.W., Berger M. (1999). Altered respiratory epithelial cell cytokine production in cystic fibrosis. J. Allergy Clin. Immunol..

[99] Nichols D.P., Chmiel J.F. (2015). Inflammation and its genesis in cystic fibrosis. Pediatr. Pulmonol..

[100] Bardin P., Sonneville F., Corvol H., Tabary O. (2018). Emerging MicroRNA therapeutic approaches for cystic fibrosis. Front. Pharmacol..

[101] Bardin P., Marchal-Duval E., Sonneville F., Blouquit-Laye S., Rousselet N., Le Rouzic P., Corvol H., Tabary O. (2018). Small RNA and transcriptome sequencing reveal the role of MiR-199a-3p in inflammatory processes in cystic fibrosis airways. J. Pathol..

[102] White N.M., Jiang D., Burgess J.D., Bederman I.R., Previs S.F., Kelley T.J. (2007). Altered cholesterol homeostasis in cultured and in vivo models of cystic fibrosis. Am. J. Physiol. Lung Cell. Mol. Physiol..

[103] Brennan S., Sly P.D., Gangell C.L., Sturges N., Winfield K., Wikstrom M., Gard S., Upham J.W., AREST C.F. (2009). Alveolar macrophages and CC chemokines are increased in children with cystic fibrosis. Eur. Respir. J..

[104] Bruscia E.M., Zhang P.-X., Ferreira E., Caputo C., Emerson J.W., Tuck D., Krause D.S., Egan M.E. (2009). Macrophages directly contribute to the exaggerated inflammatory response in cystic fibrosis transmembrane conductance regulator^−/−^ mice. Am. J. Respir. Cell Mol. Biol..

[105] Del Porto P., Cifani N., Guarnieri S., Di Domenico E.G., Mariggiò M.A., Spadaro F., Guglietta S., Anile M., Venuta F., Quattrucci S. (2011). Dysfunctional CFTR alters the bactericidal activity of human macrophages against *Pseudomonas Aeruginosa*. PLoS ONE.

[106] Painter R.G., Valentine V.G., Lanson N.A., Leidal K., Zhang Q., Lombard G., Thompson C., Viswanathan A., Nauseef W.M., Wang G. (2006). CFTR expression in human neutrophils and the phagolysosomal chlorination defect in cystic fibrosis. Biochemistry.

[107] Moss R.B., Bocian R.C., Hsu Y.P., Dong Y.J., Kemna M., Wei T., Gardner P. (1996). Reduced IL-10 secretion by CD4^+^ T lymphocytes expressing mutant cystic fibrosis transmembrane conductance regulator (CFTR). Clin. Exp. Immunol..

[108] Gao Z., Su X. (2015). CFTR regulates acute inflammatory responses in macrophages. QJM Mon. J. Assoc. Phys..

[109] Wang H., Cebotaru L., Lee H.W., Yang Q., Pollard B.S., Pollard H.B., Guggino W.B. (2016). CFTR controls the activity of NF-ΚB by enhancing the degradation of TRADD. Cell. Physiol. Biochem. Int. J. Exp. Cell. Physiol. Biochem. Pharmacol..

[110] Nakajima S., Hiramatsu N., Hayakawa K., Saito Y., Kato H., Huang T., Yao J., Paton A.W., Paton J.C., Kitamura M. (2011). Selective abrogation of BiP/GRP78 blunts activation of NF-ΚB through the ATF6 branch of the UPR: Involvement of C/EBPβ and MTOR-dependent dephosphorylation of Akt. Mol. Cell. Biol..

[111] Yu Y., Zhang L., Liu Q., Tang L., Sun H., Guo H. (2015). Endoplasmic reticulum stress preconditioning antagonizes low-density lipoprotein-induced inflammation in human mesangial cells through upregulation of XBP1 and suppression of the IRE1α/IKK/NF-ΚB pathway. Mol. Med. Rep..

[112] Chiang S.-H., Bazuine M., Lumeng C.N., Geletka L.M., Mowers J., White N.M., Ma J.-T., Zhou J., Qi N., Westcott D. (2009). The protein kinase IKKepsilon regulates energy balance in obese mice. Cell.

[113] Hu P., Han Z., Couvillon A.D., Kaufman R.J., Exton J.H. (2006). Autocrine tumor necrosis factor alpha links endoplasmic reticulum stress to the membrane death receptor pathway through IRE1alpha-mediated NF-KappaB activation and down-regulation of TRAF2 expression. Mol. Cell. Biol..

[114] Tufanli O., Telkoparan Akillilar P., Acosta-Alvear D., Kocaturk B., Onat U.I., Hamid S.M., Çimen I., Walter P., Weber C., Erbay E. (2017). Targeting IRE1 with small molecules counteracts progression of atherosclerosis. Proc. Natl. Acad. Sci. USA.

[115] Jiang H.-Y., Wek S.A., McGrath B.C., Scheuner D., Kaufman R.J., Cavener D.R., Wek R.C. (2003). Phosphorylation of the alpha subunit of eukaryotic initiation factor 2 is required for activation of NF-KappaB in response to diverse cellular stresses. Mol. Cell. Biol..

[116] Keestra-Gounder A.M., Byndloss M.X., Seyffert N., Young B.M., Chávez-Arroyo A., Tsai A.Y., Cevallos S.A., Winter M.G., Pham O.H., Tiffany C.R. (2016). NOD1 and NOD2 signalling links ER stress with inflammation. Nature.

[117] Janssens S., Pulendran B., Lambrecht B.N. (2014). Emerging functions of the unfolded protein response in immunity. Nat. Immunol..

[118] Gardner B.M., Pincus D., Gotthardt K., Gallagher C.M., Walter P. Endoplasmic Reticulum Stress Sensing in the Unfolded Protein Response. https://pubmed.ncbi.nlm.nih.gov/23388626/.

[119] Cui W., Li J., Ron D., Sha B. (2011). The structure of the PERK kinase domain suggests the mechanism for its activation. Acta Crystallogr. D Biol. Crystallogr..

[120] Carrara M., Prischi F., Nowak P.R., Ali M.M. (2015). Crystal structures reveal transient PERK luminal domain tetramerization in endoplasmic reticulum stress signaling. EMBO J..

[121] Holcik M., Sonenberg N. (2005). Translational control in stress and apoptosis. Nat. Rev. Mol. Cell Biol..

[122] Proud C.G. (2005). EIF2 and the control of cell physiology. Semin. Cell Dev. Biol..

[123] Tang A.C., Saferali A., He G., Sandford A.J., Strug L.J., Turvey S.E. (2017). Endoplasmic reticulum stress and chemokine production in cystic fibrosis airway cells: Regulation by STAT3 modulation. J. Infect. Dis..

[124] Tam A.B., Mercado E.L., Hoffmann A., Niwa M. (2012). ER stress activates NF-ΚB by integrating functions of basal IKK activity, IRE1 and PERK. PLoS ONE.

[125] Paramasivan S., Bassiouni A., Shiffer A., Dillon M.R., Cope E.K., Cooksley C., Ramezanpour M., Moraitis S., Ali M.J., Bleier B. (2020). The international sinonasal microbiome study: A multicentre, multinational characterization of sinonasal bacterial ecology. Allergy.

[126] Coburn B., Wang P.W., Diaz Caballero J., Clark S.T., Brahma V., Donaldson S., Zhang Y., Surendra A., Gong Y., Elizabeth Tullis D. (2015). Lung microbiota across age and disease stage in cystic fibrosis. Sci. Rep..

[127] Vongthilath R., Richaud Thiriez B., Dehillotte C., Lemonnier L., Guillien A., Degano B., Dalphin M.-L., Dalphin J.-C., Plésiat P. (2019). Clinical and microbiological characteristics of cystic fibrosis adults never colonized by *Pseudomonas Aeruginosa*: Analysis of the French CF registry. PLoS ONE.

[128] Lipuma J.J. (2010). The changing microbial epidemiology in cystic fibrosis. Clin. Microbiol. Rev..

[129] Stecenko A.A., King G., Torii K., Breyer R.M., Dworski R., Blackwell T.S., Christman J.W., Brigham K.L. (2001). Dysregulated cytokine production in human cystic fibrosis bronchial epithelial cells. Inflammation.

[130] Medzhitov R. (2007). Recognition of microorganisms and activation of the immune response. Nature.

[131] Moreira L.O., Zamboni D.S. (2012). NOD1 and NOD2 signaling in infection and inflammation. Front. Immunol..

[132] Chmiel J.F., Berger M., Konstan M.W. (2002). The role of inflammation in the pathophysiology of CF lung disease. Clin. Rev. Allergy Immunol..

[133] Bedi B., Maurice N.M., Ciavatta V.T., Lynn K.S., Yuan Z., Molina S.A., Joo M., Tyor W.R., Goldberg J.B., Koval M. (2017). Peroxisome proliferator-activated receptor-γ agonists attenuate biofilm formation by *Pseudomonas Aeruginosa*. FASEB J. Off. Publ. Fed. Am. Soc. Exp. Biol..

[134] Ahmadian M., Myoung Suh J., Hah N., Liddle C., Atkins A.R., Downes M., Evans R.M. PPARγ Signaling and Metabolism: The Good, the Bad and the Future. https://pubmed.ncbi.nlm.nih.gov/23652116/.

[135] Smith J.A. (2018). Regulation of cytokine production by the unfolded protein response; implications for infection and autoimmunity. Front. Immunol..

[136] Bedi B., Lin K.-C., Maurice N.M., Yuan Z., Bijli K., Koval M., Hart C.M., Goldberg J.B., Stecenko A., Sadikot R.T. (2020). UPR modulation of host immunity by *Pseudomonas Aeruginosa* in cystic fibrosis. Clin. Sci. Lond. Engl..

[137] Grootjans J., Kaser A., Kaufman R.J., Blumberg R.S. (2016). The unfolded protein response in immunity and inflammation. Nat. Rev. Immunol..

[138] Paton A.W., Srimanote P., Talbot U.M., Wang H., Paton J.C. (2004). A new family of potent AB(5) cytotoxins produced by shiga toxigenic *Escherichia Coli*. J. Exp. Med..

[139] Shenderov K., Riteau N., Yip R., Mayer-Barber K.D., Oland S., Hieny S., Fitzgerald P., Oberst A., Dillon C.P., Green D.R. (2014). Cutting edge: Endoplasmic reticulum stress licenses macrophages to produce mature IL-1β in response to TLR4 stimulation through a caspase-8- and TRIF-dependent pathway. J. Immunol..

[140] Duvigneau J.C., Luís A., Gorman A.M., Samali A., Kaltenecker D., Moriggl R., Kozlov A.V. (2019). Crosstalk between inflammatory mediators and endoplasmic reticulum stress in liver diseases. Cytokine.

[141] Dalet A., Argüello R.J., Combes A., Spinelli L., Jaeger S., Fallet M., Vu Manh T.-P., Mendes A., Perego J., Reverendo M. (2017). Protein synthesis inhibition and GADD34 control IFN-β heterogeneous expression in response to DsRNA. EMBO J..

[142] Cláudio N., Dalet A., Gatti E., Pierre P. (2013). Mapping the crossroads of immune activation and cellular stress response pathways. EMBO J..

[143] Tilney L.G., Harb O.S., Connelly P.S., Robinson C.G., Roy C.R. (2001). How the parasitic bacterium *Legionella Pneumophila* modifies its phagosome and transforms it into rough ER: Implications for conversion of plasma membrane to the ER membrane. J. Cell Sci..

[144] Hempstead A.D., Isberg R.R. (2015). Inhibition of host cell translation elongation by *Legionella Pneumophila* blocks the host cell unfolded protein response. Proc. Natl. Acad. Sci. USA.

[145] Radhakrishnan G.K., Harms J.S., Splitter G.A. (2011). Modulation of microtubule dynamics by a TIR domain protein from the intracellular pathogen *Brucella Melitensis*. Biochem. J..

[146] Shima K., Klinger M., Schütze S., Kaufhold I., Solbach W., Reiling N., Rupp J. (2015). The role of endoplasmic reticulum-related BiP/GRP78 in interferon gamma-induced persistent *Chlamydia Pneumoniae* infection. Cell. Microbiol..

[147] Webster S.J., Ellis L., O’Brien L.M., Tyrrell B., Fitzmaurice T.J., Elder M.J., Clare S., Chee R., Gaston J.S.H., Goodall J.C. (2016). IRE1α mediates PKR activation in response to *Chlamydia Trachomatis* infection. Microbes Infect..

